# Modernizing health information technology: lessons from healthcare delivery systems

**DOI:** 10.1093/jamiaopen/ooaa027

**Published:** 2020-09-03

**Authors:** Joseph Amlung, Hannah Huth, Theresa Cullen, Thomas Sequist

**Affiliations:** o1 Global Health Informatics, Center for Biomedical Informatics, Regenstrief Institute Inc., Indianapolis, Indiana, USA; o2 Indiana University, Bloomington, Indiana, USA; o3 Division of General Medicine, Department of Health Care Policy, Harvard Medical School, Brigham and Women’s Hospital, Boston, Massachusetts, USA

**Keywords:** medical informatics, health information systems, decision-making, organizational, organizational innovation, qualitative research

## Abstract

**Objective:**

To identify recurrent themes, insights, and process recommendations from stakeholders in US organizations during the health information technology (HIT) modernization of an existing electronic health record (EHR) to a commercial-off-the-shelf product in both resource-plentiful settings and in a resource-constrained environment, the US Indian Health Service.

**Materials and Methods:**

Thirteen qualitative interviews with stakeholders in various organizations were conducted about HIT modernization efforts. Using a Theory of Change framework, recurring themes were identified and analyzed.

**Results:**

The interviewees emphasized the importance of organizational and process revision during modernization, converting historical data, and clinical and leadership involvement. HIT implementation required technological and infrastructure redesign, additional training, and workflow reconfiguration. Motivations for modernization included EHR usability dissatisfaction, revenue enhancements, and improved clinical operations. Decision-making strategies, primarily during HIT selection, included meetings with stakeholders. Successful modernization resulted in improvements in clinical operations, patient experience, and financial outlay.

**Discussion:**

Existing implementation frameworks fail to provide experiential feedback, such as implementation challenges, like data conversion, regulatory, functionality, and interoperability requirements. Regardless of the healthcare environment, HIT modernization requires the engagement of leadership and end-users during HIT selection and through all stages of the implementation to prepare people, processes, and technology. Organizations must iteratively define the technological, infrastructure, organizational, and workflow changes required for a successful HIT modernization effort.

**Conclusions:**

HIT modernization is an opportunity for organizational and technological change. Successful modernization requires a comprehensive, intentional, well-communicated, and multidisciplinary approach. Resource-constrained environments have the additional challenges of financial burdens, limited staffing, and unstable infrastructure.

**Lay summary**

Health information technology (HIT) modernization involves upgrading or replacing an existing health delivery system that no longer meets an organization’s needs. Although many articles describe the process of HIT modernization, few have documented the organizational struggles and demands of a modernization effort. We identify recurrent themes, insights, and process recommendations from organizational leaders who have completed a modernization effort.

Using qualitative interviews conducted with 13 healthcare organizations, including those in resource-plentiful settings and in the resource-constrained environment of the US Indian Health Service, we found that a successful HIT modernization includes more than a technological upgrade or replacement. It should also involve improving the organization by revising the staffing structure, healthcare workflows, and more to meet the needs of the new technology. This should be done through engagement of all stakeholders, including healthcare workers, information technology staff, organizational leadership, and more.

HIT modernization is an opportunity for organizational and technological change, whether an organization has many or few resources available. However, challenges in modernization may arise when converting historical data from the previous HIT system to the new system. Understanding the organization’s regulatory, functionality, and interoperability needs are key, because HIT modernization requires a comprehensive, intentional, well-communicated, and multidisciplinary approach.

## INTRODUCTION 

With advancements in health information technology (HIT), healthcare delivery systems are modernizing existing HIT systems in hopes of improving quality and enhancing revenue.^1–3^ HIT modernization involves the improvement or replacement of an existing, typically outdated, HIT system that is unable to meet organizational, regulatory, or other requirements over the long term. The term “modernization” differs from HIT adoption, which involves the first-time implementation of an HIT system within a healthcare setting, often replacing manual and/or paper-based processes with technological solutions. Modernization, however, replaces or upgrades an existing HIT system, often with the intent of centralizing systems and enhancing technological efficiency.

Modernization is a significant undertaking, involving the investment of time, labor, and financial resources into overhauling the current HIT system or replacing it. Some healthcare organizations opt to purchase a commercial-off-the-shelf (COTS) product, while others have developed and maintained their own homegrown system. This occurs both in private healthcare systems and systems under government operation, such as the resource-constrained Indian Health Service (IHS), which provides care to nearly 2.6 million American Indian and Alaska Native people across the United States.^4^ Because IHS’ homegrown HIT system is built on the Veterans Affairs’ (VA) electronic health record (EHR) and the VA has chosen to transition to a COTS product, the IHS must create and execute a strategy to modernize its HIT.^5^ IHS offers unique insight into the struggles of modernization in more resource-constrained and rural healthcare environments with populations where social determinants of health are a focus,^6^ not unlike many low- and middle-income countries.

A literature search regarding the modernization or adoption of HIT systems produced few results with direct feedback from stakeholders that led such an effort. Most sources represented single institution experiences. Several critical factors are apparent, however, including leadership support,^7–10^ multidisciplinary teams,^11–14^ a rigorous evaluation and selection process with defined requirements,^7^^,^^8^^,^^11^^,^^12^^,^^15–21^ investing in training and support staff,^7^^,^^8^^,^^10^^,^^12^^,^^17^^,^^22–24^ and continuing a process of stabilization and optimization after go-live.^8^^,^^9^^,^^25^ Though this literature provides useful insight, it focuses largely on initial adoption of HIT, rather than modernization. A more comprehensive narrative of successful decision-making and process for modernization has been described^26^^,^^27^; however, these descriptions come only from well-resourced settings with little indication of diversity in the healthcare setting.

The Theory of Change^28^ is one approach that has been used in similar efforts where health systems attempt to implement interventions and engage stakeholders.^29^ The narrative of Theory of Change involves understanding the context in which an intervention is implemented, the intended long-term change, the process of making the change, and what assumptions will be made.^30^ Theory of Change has been used in healthcare settings to implement mental health interventions internationally^31^ and for translating evidence into priority setting,^32^ though we found no applications of the theory to HIT modernization.

We hypothesize that the Theory of Change framework can inform the approach to HIT modernization in the United States but has the potential to be leveraged internationally. We believe that this framework will promote a well-defined and successful HIT modernization effort, but this framework should be robust with respect to resource availability for any organization undergoing modernization. By interviewing healthcare institutions who successfully modernized their HIT, both in resource-constrained, strictly governed environments like IHS and in resource-plentiful and less strictly governed settings, we sought to inform guidance for successfully achieving HIT modernization.

## OBJECTIVE

To identify recurrent themes, insights, and process recommendations from stakeholders in US organizations during the HIT modernization of an existing EHR to a COTS product in both resource-plentiful settings and in a resource-constrained environment, the US Indian Health Service.

## MATERIALS AND METHODS

### Study design, population, and setting

Between May and July 2019, we conducted semi-structured qualitative interviews among a sample of healthcare provider systems that included a variety of institutions by size, geography, and other characteristics. Our goal was to identify healthcare provider systems that had transitioned to a new EHR system within the last 10 years. We opted to speak with diverse and informative organizations to identify best practices for IHS HIT modernization goals and potentially for HIT modernization efforts outside of the United States. We identified the potential healthcare systems using 3 approaches: (1) presence on the US News and World Report 2018–2019 Best Hospitals Honor Roll^33^; (2) a convenience sample of systems known to the study team that have undergone a recent transition of their EHR system; and (3) a random sample of hospitals or clinics from within the IHS that had transitioned from the existing IHS EHR to a new vendor system as of October 2018.^4^

We targeted interviewees in upper-level leadership positions within their health system who had the most direct knowledge of the HIT transition. We invited 27 healthcare systems via email, and 13 (48%) agreed to participate, each providing one interviewee who provided key leadership in the organization’s HIT modernization. Roles of interviewees varied, including Health Directors, Chief Information Officers, Chief Medical Informatics Officers, and IT Managers.

All 13 interviewees were provided with the study protocol guidelines and were ensured of their personal and institutional confidentiality. After a review of said protocols, we received verbal consent from all interviewees to obtain audio recordings of the interview. The study protocol was approved by Indiana University’s Institutional Review Board (IRB) (Protocol #1903832632).

### Data collection

A semi-structured interview guide (see Supplementary File) was developed with intent to fill in gaps left from the literature search, following the Theory of Change narratives. The interview guide contained 4 sections: (1) interviewee professional background and experience, (2) general health system characteristics, (3) HIT infrastructure within the health system, and (4) EHR conversion. For the EHR conversion, we focused on the key concepts of motivating factors for conversion, the decision-making process, the implementation of the process, measures of a successful conversion, and lessons learned from the conversion. Two team members interviewed participants using this interview guide. Interviewers were instructed to probe deeper into any topic that an interviewee could describe in more detail.^34^

### Data analysis

We identified recurring themes from the qualitative interviews to build a list of common words, phrases, and topics discussed by interview participants. We coded the transcribed interviews using Dedoose,^35^ a web-based qualitative data analysis tool. These themes were arranged into a hierarchical structure under their respective question group, and we added codes as additional themes surfaced during the data collection process. All excerpts and codes were verified by a second study team member. Organization characteristics were assigned to serve as descriptors and are shown in Table 1. We compared code counts across descriptors to qualitatively view differences between interview descriptors, as shown in Table 1. This comparison across descriptor groups allowed us to identify characteristics that may affect the motivations and outcomes discussed during interviews. We elected to conduct the interviews until new themes no longer emerged, consistent with the concept of data saturation.^36^ If new themes continued to emerge in current interviews, then more outreach was conducted to continue data collection.

**Table 1.** Interview descriptors

Interview descriptors	Groupings
Organization type	IHS-affiliated (IA) Non-IHS-affiliated (NIA)
An organization was classified as either IHS-affiliated (IA) or non-IHS-affiliated (NIA). IHS-affiliated organizations included those that operated under or in association with the Indian Health Service. Non-IHS-affiliated organizations represented all other interviewed organizations.	
Organization size	Clinic only 1 hospital 2–5 hospitals 6+ hospitals
Number of clinics or hospitals under the organization’s direct management.	
HIT type pre-transition	Homegrown COTS (commercial-off-the-shelf) Both
Before HIT modernization occurred at the organization, the organization’s HIT system may have been developed by the organization or an affiliated group specifically for that organization (homegrown) or developed by a different vendor and adapted to the organization’s needs (COTS). Some organizations utilized a combination of homegrown and COTS (both).	
Vendor size post-transition	Medium Large
After HIT modernization occurred at the organization, the new HIT system was a product developed by a vendor. Large vendors include Epic and Cerner, while Medium vendors include athenahealth, Greenway Intergy, and NextGen Healthcare.	
Transition duration	0–1 year 1–2 years 2–3 years 3+ years
Number of years between the decision to transition to another HIT system and the go-live date with that new system.	

## RESULTS

### Study population characteristics

We interviewed 13 organizations whose health systems varied by organization type, size, HIT type prior to transition, vendor size post-transition, and transition duration (Table 2). We identified 5 primary theme groups: (1) motivating factors to switch HIT systems, (2) deciding to switch and which HIT system to pursue, (3) implementation process, (4) lessons learned from switching HIT, and (5) measures of success. New themes were identified up until the seventh interview. Subsequent interviews reinforced already-established themes, indicating data saturation as asserted by Guest G., Bunce A., and Johnson L.^36^ These primary theme groups and their underlying themes were listed in Table 3 and were narratively described if they were highly discussed during interviews or if they represented novel concepts that were not addressed in the literature. Impactful quotes from interviews were listed in Table 4 with their respective theme and theme group, along with the interviewee’s role.

**Table 2.** Characteristics of interview sample

	Count (%)
Organization type	
IHS-affiliated (IA)	6 (46)
Non-IHS-affiliated (NIA)	7 (54)
Organization size	
1 hospital	2 (15)
2–5 hospitals	3 (23)
6+ hospitals	4 (31)
Clinic only	4 (31)
HIT type pre-transition	
Homegrown	8 (62)
COTS	4 (31)
Both	1 (8)
Vendor size post-transition	
Medium	4 (31)
Large	9 (69)
Transition duration	
0–1 year	5 (38)
1–2 years	4 (31)
2–3 years	1 (8)
3+ years	3 (23)

**Table 3.** Comments received by theme

Theme	Number of comments received	Theme	Number of comments received
**Motivating factors to switch HIT systems**	**93**	**Implementation process**	**103**
Cost-saving and/or revenue-enhancing	23	Time spent on implementation	20
Dissatisfaction with EHR usability	22	Speed of transition	20
Improved clinical operations or integration	19	Gradual transition	18
Connection to major vendor	11	Staffing changes	17
Improved quality and safety	10	Technology/infrastructure upgrades	17
Inadequate support from IHS	9	Training	16
Security concerns	8	Hired extra staff	12
System not optimized for billing	7	Training from vendor	11
Maintenance costs	6	Organizational restructuring	10
Want something new	5	Major changes required	10
Regulations or reporting quality measures	1	Process evaluation/change	9
**Deciding to switch and which HIT system to pursue**	**57**	Established implementation process by vendor	6
Stakeholder meetings	37	Worked with consultant	6
Leadership and end users	21	Clinical involvement	6
Leadership only	14	Minor changes required	4
RFA/RFI process	9	Training from consultants	4
Consultant or other outside party	8	Champion users	3
Cost analysis	2	Cut back on staff	3
**Measures of success**	**65**	**Lessons learned from switching HIT**	**75**
Clinical improvements	25	Advice for IHS’ HIT modernization	36
Patient improvements	23	Change the people/process, not just technology	16
Financial benefits	16	Clinical involvement	11
Non-clinical improvements	10	Home-grown system is difficult	9
Improved interoperability	4	Data conversion	8
Less in-house expertise required	3	Leadership involvement	8

**Table 4.** Interview quotes related to themes

Question group	Theme	Quote and title of interviewee (IA = IHS-affiliated organization, NIA = non-IHS-affiliated)
Motivating factors to switch HIT systems	Dissatisfaction with EHR usability	“[Our EHR] would get the job done, but there was a lot of room for improvement. It basically was slow, and there were a lot of glitches. We knew there was something better out there.” (IA5—Health Director)
	Cost-saving or revenue-enhancing	“[Our homegrown] system was built for physicians and clinicians, not optimized for billing. We had workarounds, but it was not a single integrated system.” (NIA1—Chief Medical Informatics Officer)
		“It was a costly transition, but within the first 18 months, it paid for itself through third-party revenue.” (IA1—Health Director)
	Opportunity to improve quality and safety	“Quality and the outcome for the patient are easier as well if you have the same system. The patient, administration, and the doctors are all aware of what is going on; this way you are able to know what is happening with the patient at all times.” (NIA4—Chief Medical Informatics Officer)
Making the decision to switch and selecting a product	Stakeholder meetings	“[Meeting with vendor] motivated the staff and created a collaborative decision by end users and myself.” (IA5—Health Director)
		“The leadership was a committee of the leading executives for healthcare delivery, systems stakeholders, and organizational members. There were about a dozen people at the highest level and the project was given the highest level of attention we had to offer.” (NIA3—Chief Medical Informatics Officer)
Implementation process	Technology/infrastructure upgrades	“There were big changes in the infrastructure network, servers, data centers, and end-user devices. These were all changed in a large way.” (NIA3—Chief Medical Informatics Officer)
	Staffing changes	“We hired a significant number of limited tenure employees and consultants. This helped bulk up the team, and we only released those short term employees and consultants at the end of the transition.” (NIA7—Chief Informatics Officer)
	Training	“What we do now is to give core training at the beginning and then elbow to elbow support during the go live. That way people do not learn everything and then forget it.” (NIA5—Chief Medical Informatics Officer)
Lessons learned from switching HIT systems	Change the people/process, not just technology	“This is not a technical challenge; it is a cultural transformation and needs to be treated as such.” (NIA2—Chief Informatics Officer)
	Clinical involvement	“One of our biggest lessons learned was that less clinical involvement during build of system leads to a less workable system.” (NIA1—Chief Medical Informatics Officer)
		“Involvement of the clinicians who will be using the system is vital. If you do not have their engagement from day one you have a high risk of failure.” (NIA6—Chief Medical Informatics Officer)
	Leadership Involvement	“[Leadership] needs to designate EHR implementation as the most important thing they are doing.” (NIA2—Chief Informatics Officer)
Measures of success	Clinical improvements and improved interoperability	“We can see patients, we are getting data in, we are able to record data, so this is a success. […] We still get complaints from some providers, but they would agree it is a vast improvement over what we previously had.” (IA2—Chief Financial Officer)
		“Being able to see other people’s records is much better than before. We see native and non-native patients but we send a lot of natives to [city]. There was a big benefit to using the exact same EHR as [city]; now we can see everything and all of the notes are co-mingled.” (IA4—Project Manager)
	Patient improvements	“Portal is pretty nice. Has been a big thing where 20 percent have access to it. Good response from patients who use it, and trying to get more patients to use it. […] After-visit summaries have become a lot more clear for patients to understand medications, follow-ups, etc. Got lots of positive feedback for that.” (NIA1—Chief Medical Informatics Officer)
	Financial benefits	“Switching reduced 99 percent of the user error on our part because we were no longer sending out bad bills. Third-party payers do not tell you a bill is bad; they just do not pay it. Subsequently, there is none of that now.” (IA3—IT Manager)

### Motivating factors to switch hit systems

#### Cost-saving or revenue-enhancing

Cost-saving or revenue-enhancing opportunities were also factors in HIT modernization; however, the majority of healthcare organizations cited the financial benefit as a side effect of modernization rather than a motivating factor for the transition.

#### Dissatisfaction with EHR usability

User dissatisfaction with EHR usability, often caused by a lack of interoperability, was a frequently mentioned motivating factor for change. The motivation for such interoperability differed by hospital network size, with smaller IHS-affiliated (IA) clinics desiring interoperability with local hospitals and larger non-IHS-affiliated (NIA) organizations desiring interoperability among all facilities under their management.

### Making the decision to switch and selecting a product

#### Stakeholder meetings

The majority of interviewed healthcare organizations involved a representative group of clinicians and other end-users in the decision process. Although the decision almost always reached executive board approval, their approaches were consistently reported to be driven heavily by clinician involvement and a response to facility complaints.

### Implementation process

#### Technology/infrastructure upgrades

The majority of organizations required additions or changes to their existing technology or infrastructure to meet the requirements of the new HIT system. Some required major overhauls to their technology, while others only underwent minor changes that were not disruptive. The improvement of internet capability and Wi-Fi availability, server enhancement or replacement, hardware provision for staff, and other reliability-enhancing capabilities were essential. Larger organizations often aimed to improve the connection between their facilities, in addition to improvements for individual facilities. The individual need for these changes was assessed by each organization as part of planning for HIT modernization.

#### Staffing changes

Some organizations underwent changes in staffing during the preparation for or adoption of the new HIT system. In certain cases, additional employees, such as IT staff or trainers, were hired to assist with implementation. Temporary employment of contractors or support from the HIT vendor was particularly common during the transition, with increases in the number of staff that were in some cases sustained post-deployment. In other cases, staffing was reduced as fewer employees were required to maintain the new HIT system. Staffing changes were discussed more commonly by NIA organizations. The following examples from interviewed organizations illustrate staff increases and decreases throughout modernization:

- Before transition: ∼150 IT staff—During transition: Up to 2000 transition-related staff—After transition: ∼500 IT Staff.
- During transition: 3000+ EHR-dedicated staff—Immediately after transition: ∼400 EHR-dedicated staff.

#### Training

End-user training to ensure the successful operation of the new HIT system was another common topic. The training derived either from the vendor of the new HIT system or from a consultant group that was not directly affiliated with the vendor. The vendor often provided trainers that directly trained and supported users during the transition, including before, during, and after the launch of the new HIT system. Consultants tended to not only provide training on how to use the system but also helped adjust business processes to align better with the system. A reported challenge was the continuation of patient care while training occurred. One reported approach with training was to train “champion users,” who already worked within the organization before modernization. These champions were heavily trained and assisted other users throughout modernization, providing additional and more relevant training to other users to supplement the core EHR training that they had received. Another approach involved the vendor providing large numbers of on-site trainers (eg, “One at-the-elbow person per every 3 clinicians”) to work closely with end-users during and shortly after the HIT launch.

#### Organizational restructuring

Some interviewees described restructuring their organization, including adjusting leadership models, altering clinical workgroups, and consolidating IT activities. For example, some organizations changed the HIT leadership model by appointing the chief operating officer in addition to the chief information officer to run their transition. Organizations also restructured to consolidate and centralize operations, in place of operating individually within geographic areas. Restructuring was performed for one of 2 reasons: to implement the new HIT system or to adjust for heightened integrated internal staffing needs for HIT maintenance. Such was most common among NIA organizations.

#### Process evaluation/change

During implementation, interviewees described evaluating existing processes to identify where changes were necessary prior to system “go-live.” While some described this step as necessary for the implementation to succeed, others described the implementation as an opportunity to optimize processes, to not only align with the new system but also to improve organizational efficiency or effectiveness. This was described particularly by NIA organizations.

### Lessons learned from switching hit systems

NIA organizations tended to emphasize changing people and processes, clinical involvement, and transparency, while IA organizations discussed the issues of data conversion and maintaining a homegrown system more frequently.

#### Change the people/process, not just technology

Interviewees felt that to achieve optimal benefit during a technology transition, the culture and processes must also be addressed. Among larger hospital systems, this included workflow standardization across all functions and departments so that the new HIT system would consistently sync with all processes in place at all healthcare facilities. This topic was discussed by NIA organizations and those that transitioned away from a homegrown system.

#### Difficulties of a homegrown system

Although homegrown systems offered the advantage of meeting an organization’s unique needs, interviewees reported that it was difficult to keep the system integrated and functionally relevant to clinical and non-clinical staff. Regulatory requirements provided another layer of challenge in maintaining the system. This topic was discussed by IA organizations and organizations that transitioned away from homegrown systems.

#### Clinical involvement

Interviewees believed that clinical staff must also be involved throughout the implementation process. Since clinical staff members are the primary users who will carry out processes on the new HIT system, a strong connection with this group should be developed and maintained to help to set expectations and identify potential risks that must be mitigated before implementation can be completed.

#### Data conversion

Data conversion was presented as a challenge for organizations that required historical data to be transferred to their new HIT system. Interviewees were particularly concerned with the process of determining which data to transfer. This challenge may lead to delays in implementation if it is not considered from the start. Interviewees reported that clinical and administrative requirements must be identified to understand which data will be required, how far back the patient history must go, and in what format the data must be converted. Data conversion was discussed primarily among IA organizations and organizations that moved away from a homegrown HIT system.

#### Leadership involvement

Some organizations stated the necessity of having the full engagement of leadership throughout the decision and implementation processes. Without guidance from leadership, the HIT system transition might stray from organizational goals and create conflict once modernization was completed. This topic was most discussed by organizations moving away from homegrown systems and by those who underwent longer HIT transitions.

### Measures of success

Clinical and patient improvements were the most commonly cited indicators of success, followed by financial benefits, non-clinical improvements, and improved interoperability. However, interviewees did not report specific methods of measurement for these indicators.

#### Clinical improvements and improved interoperability

Clinical end-users generally noticed improvements after the HIT switch, attributed primarily to improved integration and interoperability. Real-time patient information became readily available among all departments and facilities, reducing inefficiencies for patients that visit multiple healthcare locations. This was discussed most by IA interviewees, those transitioning away from homegrown systems, and those transitioning to a medium-sized vendor.

#### Other improvements

Patients noticed an improvement after the HIT switch both directly (eg, improved patient portal or other patient-facing technologies) and indirectly (eg, improved interactions between providers and HIT). Financial benefits were also experienced due to increased efficiency of financial operations by providing smoother billing and claims processing and due to less requirement for in-house HIT support personnel to maintain the new HIT system.

## DISCUSSION

HIT modernization, especially for larger healthcare organizations, can be a disruptive and costly undertaking, though it can benefit the organization. Given the lack of published literature on the topic, we conducted a qualitative study of 13 interviews from organizations that had completed HIT modernization in the last 10 years, representing delivery systems across the US in IA and NIA settings. Despite the variety of organizations with respect to size, EHR systems, and availability of resources, the interviews yielded consistent themes regarding the process of modernization, lessons learned, and success experienced in healthcare operations. During interviews regarding HIT modernization, new themes stopped appearing after the seventh interview, indicating that data saturation was achieved for this interview guide.

In agreement with published literature, interviewees emphasized that leadership must be unconditionally engaged in HIT modernization to set expectations and communicate the value of modernization to all stakeholders, especially end-users.^7–10^ They also agreed that it was best to utilize multidisciplinary teams to plan and carry out modernization efforts with clinical representation as core members.^11–13^ Interviewees heavily described executing implementation and training plans, which varied by organizational needs but often included user training, system customizations, and hardware/networking adjustments.^8^^,^^21–23^ Launching the new HIT system by adding staff specifically for training and development, along with system stabilization and ongoing evaluation,^8^^,^^13^^,^^25^ were key discussion points in interviews.

While interviewed organizations were of varying characteristics, IA and NIA organizations appeared to vary the most in their discussed topics. Differing motivations for and approaches to modernization between the resource-strained IA organizations and the larger, more resource-available NIA organizations were noted. Many motivations and implemented changes were related to meaningful use, particularly for IA organizations, but NIA organizations seized the opportunity for improving the organization as a whole. At a high level, IA organizations primarily focused on technology improvements, with little emphasis on organizational or process changes, while NIA organizations modernized more than their technology, taking the opportunity to change their organization as a whole. The lack of funding for IA organizations may have contributed to technology and infrastructure being the most important factors to modernize, but the small size of the healthcare sites allowed for easier changes to people and processes where needed. NIA organizations, however, often were already well-equipped in terms of technology and infrastructure, but their large size introduced more complexity when changing people and processes. Hence, they seized the opportunity to change more than the technology, possibly because the opportunity to make such a change is uncommon, given the cost of doing so.

While these findings showed some conformity with existing frameworks and theories, such as Organizational Change Theory^37^ and Implementation Science,^38^ they contain some more unique aspects. Theory of Change more effectively facilitated the approach of understanding the contexts and motivations behind HIT modernization, identifying assumptions, and planning out the steps required to modernize. In particular, modernization is a context- and assumption-driven effort. For example, the difficulty in maintaining homegrown HIT systems is often overlooked, although it can be a major driving factor in modernization. Keeping up with regulatory, functionality, and interoperability-related requirements can be a monumental task. Data conversion is also a major element in modernization and may be a deciding factor in updating a homegrown system as opposed to adopting a COTS system instead. Colicchio et al.,^26^ in particular, similarly describes modernization from a homegrown system and the effects of incomplete data conversion. This study emphasizes several other topics described in our interviews, including staff changes and organizational restructuring. Kiepek and Sengstack^27^ also acknowledge the importance of on-site support for end-users during the transition but do not discuss the preparatory work to get to the actual implementation. Overall, our findings support claims from these studies but broaden the scope with respect to the variety in healthcare settings and resource availability.

Despite seeing some similar findings in the literature, these interviews revealed deeper insights and key guidance for HIT modernization. While all phases of modernization are necessary to some extent, the immediate preparations, especially the implementation process, appear to be the most difficult. In particular, organizations must understand the details of data conversion early in the process, ensuring that end-users can define what information is required to succeed and where areas of compromise will be needed. This is particularly necessary for those transitioning from a homegrown HIT system. When preparing for modernization and selecting an HIT solution, the involvement of clinicians is necessary to ensure trust and meaningful use of the system. While there are various engagement techniques, stakeholder engagement is a valuable step for reducing the risk of failure and fostering ownership in modernization by involving end-users before undertaking modernization and selecting a product. All leadership must be heavily engaged throughout modernization,^6–8^ rather than solely involving a Chief Information Officer. These findings support and expand upon the Waterfall Software Development Model.^39^

The HIT implementation process is a complex journey, with many approaches tailored to the specific needs of the organization. Regardless of the setting, modernization requires preparation with infrastructure, training and involvement of staff, and continual end-user and leadership engagement. Organizations must define the scope of changes required to accommodate the new HIT with respect to technology, infrastructure, processes, and organizational structure. These changes must be understood by end-users and leadership alike. Training approaches should align with the organization’s needs and culture to educate not only how to incorporate the new HIT system into their workflow, but also how to improve the workflows themselves.

From this study, we suggest several best practices for HIT modernization, which can apply both in US and international settings. When implementing an HIT modernization project, organizations should consider not only the technology to be selected, but should capitalize on the opportunity to align people, processes, and technology and transform their healthcare to yield major efficiencies, rather than simply keeping up with regulatory or other requirements. Additionally, an often unexpected challenge in modernization is data conversion and the gravity of not having historical data present in the new HIT system. This must be accounted for early in the modernization process. Success can be measured in many ways, including clinician and patient satisfaction and financial benefit upon switching HIT systems. However, a commonly missed opportunity in HIT modernization is the establishment of measurable success indicators. There can also be extreme difficulty for an organization to develop and maintain its own HIT systems, which often leads organizations away from keeping their homegrown systems, despite the benefits that they may bring. Kiepek and Sengstack^27^ suggest some potential success indicators.

### Limitations of study

Our study is among the first to evaluate HIT modernization in diverse settings; however, it does have limitations. We employed a convenience sample with the intent of identifying best practices, rather than representing all healthcare organizations. Rather than selecting a single role (eg, a health director), various roles were allowed in this study, although interviewees of varying roles may have offered different perspectives. We had no method of verifying success for an organization’s modernization effort, which relates to the lack of a standard measure of success. Finally, and possibly most importantly, interviewees from IA organizations may have a personal stake in these interviews, given the completed and impending changes in HIT infrastructure within their organization.^40^

## CONCLUSION

HIT modernization is an important, challenging, and risky undertaking for any healthcare organization. We aimed to guide organizations through the entire modernization process in a variety of settings with support from a Theory of Change approach. While HIT modernization can be successful solely by improving an organization’s technology, it is best treated as an opportunity for organizational change in addition to technological change, particularly for larger, more complex organizations. Successful modernization of a HIT system, regardless of size and complexity, requires a comprehensive, intentional, well-communicated, and multidisciplinary approach. Further research is necessary to validate and better understand these best practices in preparing for modernization, however, by focusing on how organizations should define requirements, select HIT solutions, and determine measurable indicators of success for HIT modernization.

## FUNDING

This project was supported by the Department of Health and Human Services as part of the HHS/IHS HIT Modernization Project (18-233-SOL-00773-RFQ1316210).

## AUTHOR CONTRIBUTIONS

JA and HH wrote the manuscript and collected interview data, and JA performed the analysis. TC and TS wrote the Interview Guide and provided guidance on the study and for the manuscript. All authors reviewed the final manuscript and are accountable for this work.

## Supplementary Material

ooaa027_Supplementary_Datat
