# Supplementary material for: Modernizing health information technology: lessons from healthcare delivery systems
Source: JAMIA Open. 2020 Sep 3;3(3):369–77. doi: 10.1093/jamiaopen/ooaa027 (PMC7660948; doi:10.1093/jamiaopen/ooaa027)
Supplement: ooaa027_Supplementary_Datat [file ooaa027_supplementary_datat.docx]

# SUPPLEMENTARY FILE - INTERVIEW GUIDE

**IHS Health Information Technology Assessment: Site Interview Guide**

**Date: _________________________**

**Site: ________________________________**

**Title: ________________________________**

**Interviewer: ________________________________**

Thank you for taking the time to talk with me today. As you know, the Department of Health and Human Services and the Indian Health Service is collaborating with the Regenstrief Institute to evaluate the current state of health information technology and make recommendations for future directions. This is part of a larger strategic project designed to inform the Indian Health Service on specific issues such as whether to invest in upgrading its current electronic health record versus partner with an external vendor to purchase and install a new electronic health record.

Before we begin, I want to tell you that this project has been approved by the Regenstrief Institute IRB. All of your responses will be treated as confidential information. We will not disclose your responses in connection with your name or the name of your facility. The interview will be recorded and transcribed so that members of our project team can review and analyze it. Any use of your responses in project reports will be de-identified, pooled with other responses, or quoted in a manner that minimizes the possibility that an individual or facility can be identified. The recordings will be destroyed at the completion of the project.

Do we have your permission to record the interview?

Do you have any questions before we begin?

**Section I Respondent Background**

1. Can you state your title/position here at your organization?
2. Can you describe your own educational or training background as it relates to your current professional activities? [eg, medical school, business school, etc]
3. Please describe your professional role(s) at this facility in more detail.
   1. How long have you been working in this role at this facility?
   2. What is your experience in health information technology applications?
4. Please describe how your role intersects with the management of health information technology resources in your organization?
   1. Electronic health record
   2. Other patient facing tools (e.g., telemedicine)
   3. Health IT infrastructure such as equipment, security, etc

**Section II Health System Background**

1. We want to collect some basic information about your health system. If you do not have this information today, it is ok to get back to us with the details.
   1. How many hospitals are included in your system?
   2. How many ambulatory or outpatient clinics are in your system?
   3. How many doctors practice in your system?
   4. Does your system include an Academic Medical Center?
   5. What care settings are included in your system – Hospital, Ambulatory, Post-Acute?

**Section III Health Information Technology Background**

1. Can you describe your organization’s previous electronic health record?
   1. Did the system use an integrated, single electronic health record across all hospitals/clinics and care settings (inpatient, outpatient, ED, etc)? Or were there multiple EHRs?
   2. Was the EHR ‘home grown’ or a vendor system? What was the name of the EHR?
   3. How many years had the EHR(s) been in use within your system prior to its replacement?
2. Can you describe the governance and management of your prior EHR?
   1. Was the system and support team under direct management of CIO (IS leadership) or CHIO (Medical leadership)?

**Section IV Electronic Health Record Conversion**

1. What year did the EHR conversion begin (not the decision process, but the actual implementation)?
2. What was your involvement in the decision process to convert the existing EHR?
   1. How would you describe your level of enthusiasm for participating before the process started?
3. What were the motivating factors in deciding to convert the existing EHR?
   1. Dissatisfaction with EHR usability?
   2. Cost-saving opportunity or revenue enhancing opportunity?
   3. Security concerns or other worries about ongoing maintenance of system?
   4. Improved clinical operations or integration across your own health system or with other health systems?
   5. Opportunity to improve quality and safety?
4. Please describe your organization’s process for deciding whether to upgrade the existing EHR or replace with a vendor system.
   1. Was there a leadership committee? If so, who were the stakeholders?
      - Were providers and/or end users engaged?
   2. Was this a board-level decision?
   3. How long did the process take from start to finish?
   4. Did you have a formal RFA process for external vendors?
   5. What EHR solution did you ultimately choose?
5. What was required to successfully complete your EHR conversion or upgrade?
   1. How long did implementation take from start to finish?
   2. Did you work with an outside consultant?
   3. What is your ideal and actual ratio of transition focused staff to number of clinicians or health care team members?
   4. Did you have to hire extra staff, or were you able to cut back on staff? How long did you require extra staff to remain in place?
   5. Did implementation require any organizational restructuring?
   6. Did implementation require significant upgrades to your information technology infrastructure?
6. Do you have a process for evaluating whether your EHR conversion achieved the desired outcomes?
   1. How successful or unsuccessful you think the EHR conversion was? What makes you think that?
   2. Are there any experiences that you would not repeat?
   3. What types of activities were the hardest to employ?
   4. What is working better than before? What is not working as well as before?
   5. Were there differences in providing and obtaining IT support before and after the switch?
7. Have patients reported to any noticeable changes in their health care experience that you feel are attributable to the new EHR?

**Section V Conclusion**

I would like to conclude our interview with some summary questions.

1. What was the most important thing you learned from you EHR conversion?
2. In retrospect, do you think the EHR conversion was “the right choice”?
3. Do you think the EHR conversion helped your organization advance its mission?
